# Norepinephrine Enhances Aerobic Glycolysis and May Act as a Predictive Factor for Immunotherapy in Gastric Cancer

**DOI:** 10.1155/2021/5580672

**Published:** 2021-03-27

**Authors:** Yangyang Wang, Shuchang Wang, Qin Yang, Jun Li, Fengrong Yu, Enhao Zhao

**Affiliations:** ^1^Department of Gastrointestinal Surgery, Renji Hospital, School of Medicine, Shanghai Jiao Tong University, Shanghai 200127, China; ^2^State Key Laboratory of Oncogenes and Related Genes, Shanghai Cancer Institute, Renji Hospital, School of Medicine, Shanghai Jiao Tong University, Shanghai 200240, China

## Abstract

**Methods:**

Monoamine neurotransmitters were detected in gastric cancer tissue and paired normal tissue, and The Cancer Genome Atlas was used to identify differentially expressed norepinephrine-degrading and synthetic enzymes. Quantitative real-time PCR and the Seahorse assay were used to determine the effect of norepinephrine on gastric cancer cell glycolysis. MAOA expression in cancer tissues was analyzed by immunohistochemistry and was compared with the patient SUVmax value of PET-CT and other clinicopathological characteristics.

**Results:**

The norepinephrine levels were markedly high in gastric cancer tissue, while the norepinephrine-degrading enzymes MAOA and MAOB showed low expression. High norepinephrine levels were associated with activated glycolysis. The MAOA or MAOB expression levels in tumor tissue were closely correlated with the patient SUV max values of PET-CT and immunotherapy evaluation indices, such as PD-L1 and the microsatellite status.

**Conclusions:**

Norepinephrine shows relatively higher expression in gastric cancer tissue than in normal tissue, and its expression level is associated with the glycolysis levels in patients. The norepinephrine-degrading enzymes MAOA and MAOB have significant expression differences in cancer and normal tissue, and their missing or low expression may predict immune therapy outcomes for gastric cancer patients. High norepinephrine levels with metabolic abnormalities may be more suitable for metabolic targeted therapy or immunotherapy.

## 1. Introduction

According to previous studies, neurotransmitters have a close relationship with the diverse malignant phenotypes of human cancers. The richness of neurotransmitters in the gastrointestinal tract tissue is involved in the occurrence and development of gastrointestinal tumors [1–3]. In the present study, the relationship between norepinephrine (NE) and glycolysis in patients, as well as that between NE and immune characteristics, was explored.

Tumor cell metabolism is different from that in normal cells because the tumor microenvironment presents hypoxia and low pH. Because malignant tumors require rapid proliferation with an energy supply, anaerobic glycolysis provides proliferation cell energy and essential products for cell division and proliferation. Tumor cell glycolysis in an aerobic environment is also known as the Warburg effect, the special metabolism of tumor cells in the tumor microenvironment [4, 5].

PD-L1 expression, the EBV infection status, the microsatellite status, and the tumor mutation burden are associated with the results of anti-PD-1 therapy based on previous studies [6, 7]. The application of immunotherapy in gastric cancer is becoming increasingly important, with significant survival benefits. Immunotherapy has also become an important treatment other than surgical treatment, chemotherapy, and targeted therapy for advanced gastric cancer patients.

To explore the differential expression of monoamine neurotransmitters in the tumor and normal tissues of gastric cancer patients, we investigated the differential expression of NE and its degrading enzyme monoamine oxidase. The expression level of the monoamine neurotransmitter NE showed a close correlation with glycolytic activity. The monoamine oxidase A (MAOA) expression level may also predict the immune therapy response to some extent. However, its function and mechanism in gastric cancer must be further explored in the future.

## 2. Materials and Methods

### 2.1. Immunohistochemical Staining and Scoring

All of the tissue samples were fixed in phosphate-buffered neutral formalin, embedded in paraffin, and then cut into 5 *μ*m thick sections. Immunohistochemistry (IHC) was performed on the sections using antibodies specific to MAOA (Proteintech) and monoamine oxidase B (MAOB) (Proteintech). Briefly, tissue sections were deparaffinized, rehydrated with graded ethanol, incubated with 0.3% hydrogen peroxide for 30 minutes, and blocked with 10% BSA (Sangon, Shanghai, China). Slides were firstly incubated using the antibody at 4°C overnight and then labeled with the HRP second antibody (Thermo Fisher Scientific, Inc., US) at room temperature for 1 h. Positive staining was visualized with DAB substrate liquid (Gene Tech, Shanghai) and counterstained with hematoxylin. All the sections were observed and photographed with a microscope (Carl Zeiss, Germany).

Scoring was conducted according to the ratio and intensity of positive-staining cells, and the scores were determined independently by two senior pathologists. The scoring by the pathologists was done in a blinded manner [8].

### 2.2. HPLC Analysis

Neurotransmitters were extracted from human gastric cancer tissues and paired normal tissues with perchloric acid. Dopamine (DA), norepinephrine (NE), epinephrine (E), and serotonin (5-HT) were analyzed by High Performance Liquid Chromatography (HPLC) [9]. Neurotransmitters concentrations were calculated with a Hewlett Packard integrator.

### 2.3. Cell Culture and Reagents

The human gastric cancer BGC-823 and HGC-27 cell lines were all maintained in the Shanghai Cancer Institute, Renji Hospital. All cells were cultured in RPMI-1640 medium (Beijing Solarbio Science & Technology Co., Ltd.) or F-12 Medium (Gibco; Thermo Fisher Scientific, Inc.) according to ATCC protocols and supplemented with 10% (*v*/*v*) fetal bovine serum (FBS) and 1% antibiotics (100 *μ*g/ml streptomycin and 100 U/ml penicillin) at 37°C in a humidified incubator at 5% CO_2_. The cell medium was replaced every 2-3 days, and 1X PBS was used for cell washing prior to the medium being replaced [10]. The concentration of norepinephrine treated above cells is 100 nmol in this study.

### 2.4. Reverse Transcription-Quantitative Polymerase Chain Reaction (RT-qPCR)

The thermocycling conditions were as follows: 60°C for 34 sec and 95°C for 15 sec for 40 cycles. Total RNA was extracted from gastric cancer tissues or gastric cancer cell lines using TRIzol reagent (Thermo Fisher Scientific, Inc.) and reverse transcribed using a PrimeScript RT-PCR kit (Takara Bio., Inc., Otsu, Japan), according to the manufacturer's protocols. RT-qPCR was performed with SYBR Premix Ex Taq (Takara Bio., Inc.) using a 7500 Real-time PCR system (Thermo Fisher Scientific, Inc.). The 2-*ΔΔ*Cq method (15) was used to quantify relative gene expression, which was normalized to *β*-actin [10]. Primers used in this study can be found in Supplementary Table 1.

### 2.5. TCGA Database and Gene Set Enrichment Analysis

For 450 GC samples in TCGA database (https://cancergenome.nih.gov/), NE synthesis and degradation enzyme mRNA expression were analyzed in 415 tumor tissue samples and 35 normal tissue samples. And 32 paired tumor tissues and normal tissues were also analyzed for NE synthesis and degradation enzymes mRNA expression. We analyzed the Top 100 cases with the highest mRNA expression and 100 cases with the lowest mRNA expression of MAOA and MAOB through using Gene Set Enrichment Analysis (GSEA, http://www.gsea-msigdb.org/gsea/index.jsp) to find a differential signal pathway.

### 2.6. ECAR and OCR Measurement in Seahorse Experiment

In vitro cell metabolic alterations were monitored with the Seahorse XF96 Flux Analyzer (Seahorse Bioscience), according to the manufacturer's instructions. BGC-823 cells were seeded in a XF96-well plate and followed by PBS and NE stimulation for 24 hr. For assessment of the real-time glycolytic rate (ECAR), an indicator of net proton loss during glycolysis, cells were incubated with unbuffered medium followed by a sequential injection of 10 mM glucose, 1 mM oligomycin (Sigma-Aldrich), and 80 mM 2-deoxyglucose (2-DG, Sigma-Aldrich, D8375). The mitochondrial respiration (OCR) was assessed using sequential injection of 1 mM oligomycin, carbonyl cyanide 4-(trifluoromethoxy)phenylhydrazone (FCCP, Sigma-Aldrich, C2920), and 2 mM antimycin A and rotenone (Sigma-Aldrich). To achieve maximal OCR, antimycin A and rotenone were used to inhibit mitochondrial respiration by blocking complex III (ubiquinone: cytochrome b-c complex). Both ECAR and OCR measurements were normalized to total protein content and reported as pmoles/min for OCR and mph/min for ECAR [11].

### 2.7. Glucose Uptake and Lactate Production Measurement

Briefly, cells were seeded into 6-well plate culture dishes and starved for 24 hr after cells were adhered. Then cells were treated with indicated antagonists for 2 hr, followed by stimulation with NE (10 mM) for additional 24 hr. The culture medium was collected for the measurement of glucose and lactate concentration, and cells were harvested for protein lysates. The glucose uptake assay was performed using a glucose assay kit (Sigma-Aldrich). Glucose consumption was calculated by deducting the measured glucose concentration in the medium from the original glucose concentration. Lactate production in the medium was detected by using the Lactate Assay Kit (BioVision, Mountain View, CA) according to the manufacturer's instruction. Results were normalized on the basis of the total protein concentration of each sample [11].

Written informed consent was obtained from all enrolled patients prior to their inclusion in the study, and the studies were conducted in accordance with Declaration of Helsinki ethical guidelines. The present study was approved by the Ethics Committee of Renji Hospital, Shanghai Jiao Tong University School of Medicine.

## 3. Statistical Analysis

Statistical analyses were done using SPSS 22.0 for windows (IBM). The chi-square test and Student's *t*-test were used for comparison between groups. Values of *p* < 0.05 were considered statistically significant.

## 4. Results

### 4.1. NE Is Highly Expressed in Gastric Cancer Tissue

**Figure 1:**
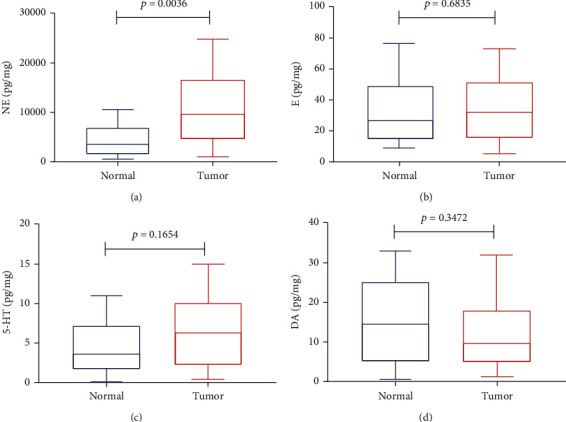
NE is more highly expressed in gastric tumor tissue than in normal tissue, while the other monoamine neurotransmitters E 5-HT and DA are not significantly different.

To identify the differential monoamine neurotransmitter expression in gastric cancer tissue and normal tissue, the neurotransmitters E, NE, DA, and 5-HT were analyzed in 15 gastric cancer patients who had undergone surgery at Renji Hospital, School of Medicine, Shanghai Jiao Tong University. The above neurotransmitter expression level was tested using HPLC analysis. NE was more highly expressed in gastric cancer tissue than in normal tissue (*p* = 0.0036) (Figure 1(a)), while the other neurotransmitters, E (*p* = 0.6835), 5-HT (*p* = 0.3472), and DA (*p* = 0.1654), showed no significant difference in expression in tumor and normal tissue (Figures 1(b)–1(d)).

### 4.2. Monoamine Neurotransmitter-Degrading Enzymes MAOA and MAOB Show Low Expression in Gastric Cancer Tissue

**Figure 2:**
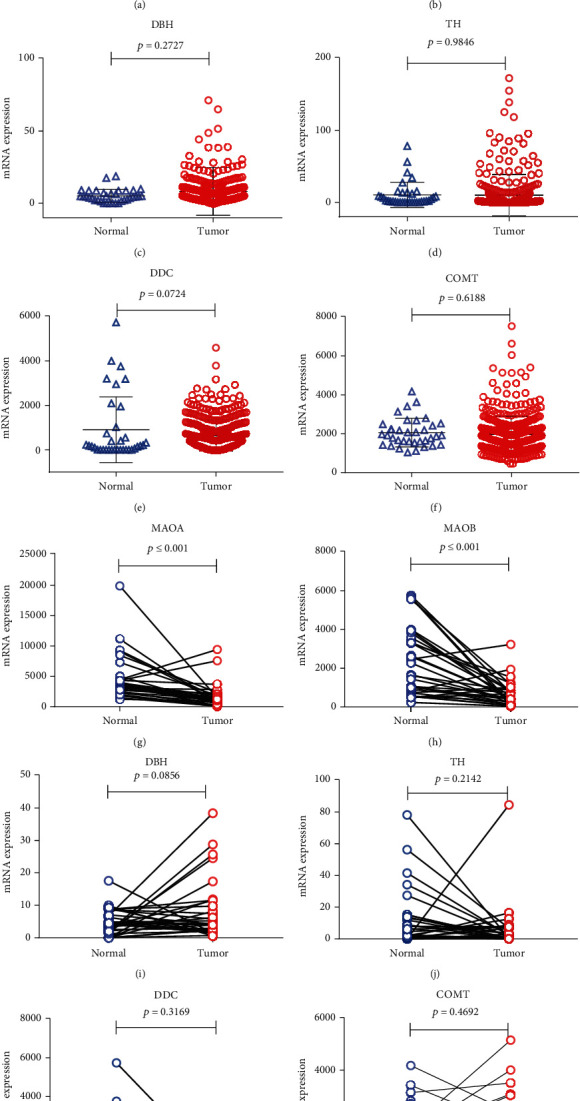
The mRNA levels of the monoamine neurotransmitter-degrading enzymes MAOA and MAOB are low in gastric cancer tissue, while those of the other synthetase and degrading enzymes TH, DDC, DBH, and COMT are not significantly different.

To further elucidate NE synthase and degrading enzyme expression in tumor and normal tissues, 415 gastric cancer tissues and 35 normal gastric tissues were analyzed in TCGA database. The MAOA (*p* ≤ 0.001) and MAOB (*p* ≤ 0.001) mRNA levels were significantly higher in normal tissues than in tumor tissues (Figures 2(a) and 2(b)). No significant difference was found regarding the expression of the other NE synthase and degrading enzymes DBH (*p* = 0.0724), TH (*p* = 0.9846), DDC (*p* = 0.0724), and COMT (*p* = 0.6188) in gastric cancer and normal tissue (Figures 2(c)–2(f)). Subsequently, we analyzed 32 paired tumor-normal tissue samples for NE synthase and degrading enzyme mRNA expression in TCGA database, and the MAOA (*p* ≤ 0.001) and MAOB (*p* ≤ 0.001) mRNA levels showed significantly higher expression in normal tissues than in the paired tumor tissues (Figures 2(g) and 2(h)). For the other NE synthase and degrading enzyme DBH (*p* = 0.0856), TH (*p* = 0.2124), DDC (*p* = 0.3169), and COMT (*p* = 0.4692) expression in gastric cancer and normal paired tissue, there is no significant difference (Figures 2(i)–2(l)).

### 4.3. MAOA and MAOB Expression Shows a Close Relationship with Glycolysis and Immune-Related Signaling Pathways

**Figure 3:**
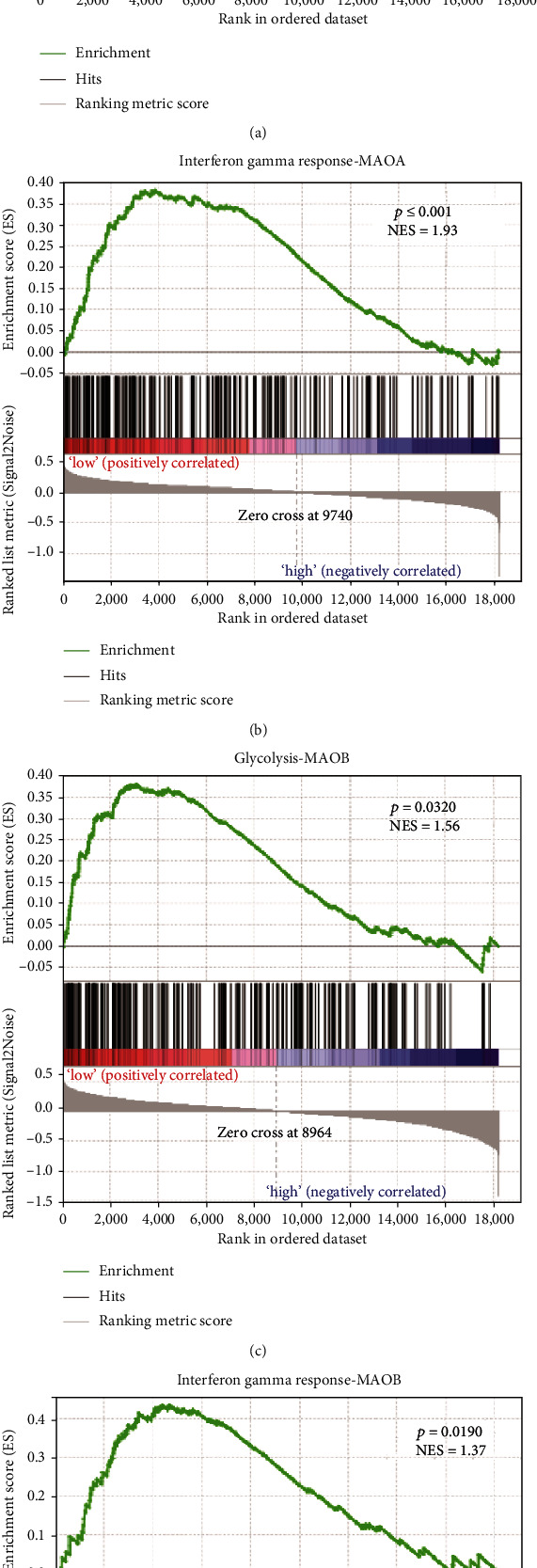
The expression levels of both MAOA and MAOB show a negative correlation with the glycolysis pathway. For the immune-related interferon gamma signaling pathway, the expression levels of both MAOA and MAOB showed a negative correlation.

Next, we further mined TCGA database for MAOA- and MAOB-related signaling pathways by applying GSEA. We compared the top 100 cases with the highest mRNA expression and 100 cases with the lowest mRNA expression of MAOA and MAOB using GSEA to identify differential signaling pathways in their highest and lowest groups. For MAOA, the glycolysis pathway (*p* ≤ 0.001) and interferon-*γ* pathway (*p* ≤ 0.001) were significantly different in the high- and low-expression groups (Figures 3(a) and 3(b)). Key glycolysis pathway and interferon-*γ* pathway genes were highly expressed or activated in the MAOA low-expression group. MAOB analysis displayed the same result and trend as MAOA analysis, with the glycolysis pathway (*p* = 0.0320) and interferon-*γ* pathway (*p* = 0.0190) showing significant differences (Figures 3(c) and 3(d)).

### 4.4. NE Promotes Tumor Cell Glycolysis

**Figure 4:**
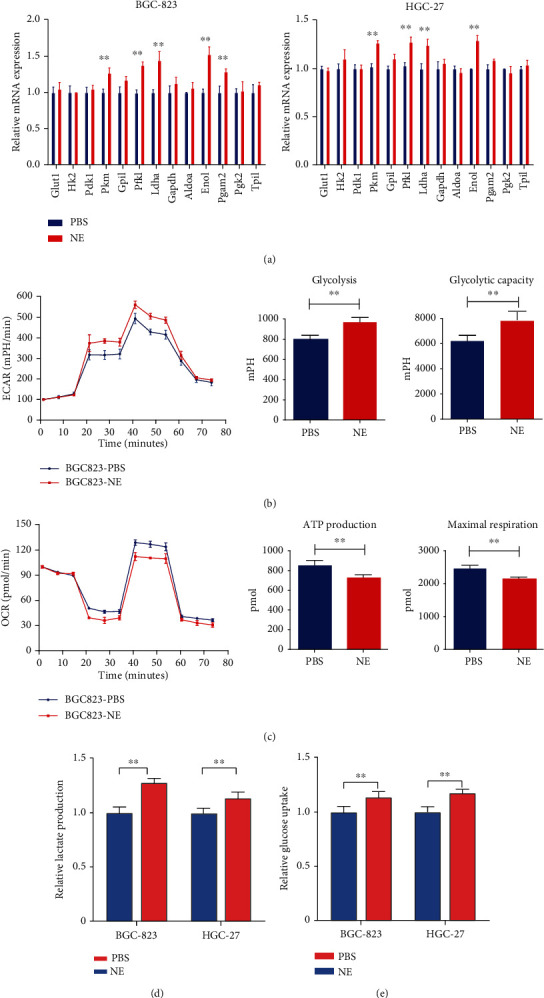
NE expression promotes tumor cell glycolysis. Regarding the gastric cancer cell line BGC-823, the Pkm, Pfkl, Ldha, Eno1, and Pgam2 mRNA expression levels were significantly elevated in the NE treatment group compared with the PBS treatment group. Regarding the other HGC-27 cancer cell line, the Pkm, Pfkl, Ldha, and Eno1 mRNA expression levels were significantly elevated in the NE treatment group compared with the PBS treatment group (a). After NE treatment, the EACR increased obviously for BGC-823 cells compared with PBS treatment. Regarding glycolysis and the glycolytic capacity, the NE and PBS treatment groups showed significant differences (b). After NE treatment, the OCR decreased for BGC-823 cells compared with that after PBS treatment. Regarding ATP production and maximal respiration, the NE and PBS treatment groups showed significant differences (c). In both BGC-823 and HGC-27 cells, lactate production and glucose uptake capability were more enhanced in the NE treatment group than in the PBS treatment group (d, e).

To investigate the effect of NE on tumor cell glycolysis, the mRNA expression levels of key glycolytic enzymes were tested after exposure to PBS and NE. Regarding the gastric cancer cell line BGC-823, the Pkm, Pfkl, Ldha, Eno1, and Pgam2 mRNA expression levels were significantly elevated in the NE treatment group. Regarding the other HGC-27 cancer cell line, the Pkm, Pfkl, Ldha, and Eno1 mRNA expression levels were significantly elevated in the NE treatment group (Figure 4(a)). Next, the Seahorse assay was performed to test the effect of NE on the ECAR and OCR in tumor cells. After NE treatment, the EACR increased and the OCR decreased for BGC-823 cells compared with PBS treatment. Concerning glycolysis, the glycolytic capacity, ATP production, and maximal respiration, the NE and PBS treatment groups showed significant differences (Figures 4(b) and 4(c)). Subsequently, lactate production and glucose uptake were detected in BGC-823 and HGC-27 cells treated with PBS and NE. In both BGC-823 and HGC-27 cells, lactate production and glucose uptake were more enhanced in the NE treatment group than in the PBS treatment group (Figures 4(d) and 4(e)).

### 4.5. MAOA and MAOB Expression Is Negatively Correlated with Glycolysis Activity

**Figure 5:**
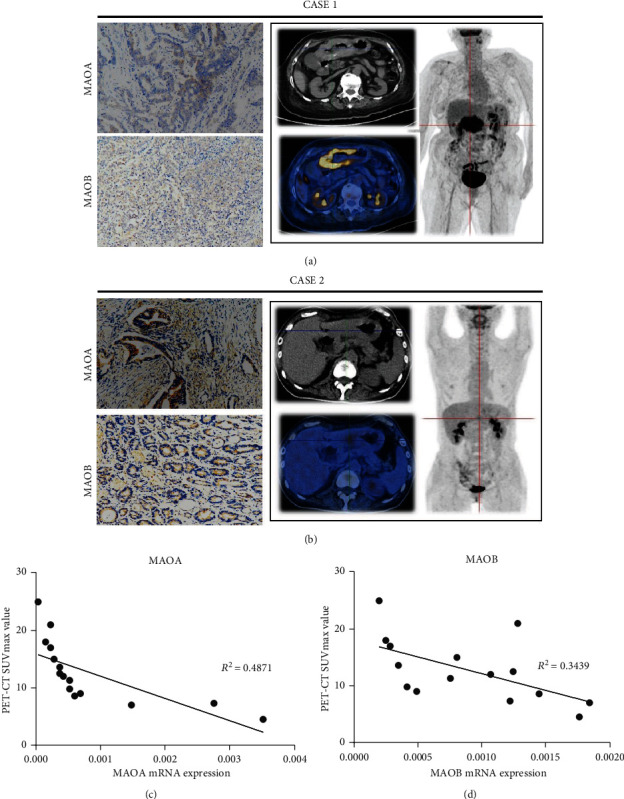
The MAOA and MAOB expression levels are negatively correlated with glycolysis activity. One case showed low MAOA and MAOB expression with high glycolysis activity (a). Another case showed high MAOA and MAOB expression with low glycolysis activity (b). MAOA mRNA expression was negatively correlated with the SUVmax value of PET-CT (c). MAOB mRNA expression was negatively correlated with the SUVmax value of PET-CT (d).

Given that PET-CT of gastric cancer patients could represent glycolysis activity in patients, we evaluated MAOA and MAOB expression using patient PET-CT graphics. We found that Patient CASE 1, with a high SUVmax value and glycolysis, showed low MAOA and MAOB expression in the tumor tissue. Patient CASE 2, who had a low SUVmax value and glycolysis, showed high MAOA and MAOB expression in the tumor tissue (Figures 5(a) and 5(b)). To further illustrate the correlation between MAOA and MAOB expression and the PET-CT SUVmax value, we evaluated 15 patients with preoperative PET-CT. The mRNA expression levels of both MAOA (*R*^2^ = 0.4871) and MAOB (*R*^2^ = 0.3439) were negatively correlated with the PET-CT SUVmax value (Figures 5(c) and 5(d)).

### 4.6. Correlation between MAOA Expression and the Patient Clinicopathological Characteristics

**Figure 6:**
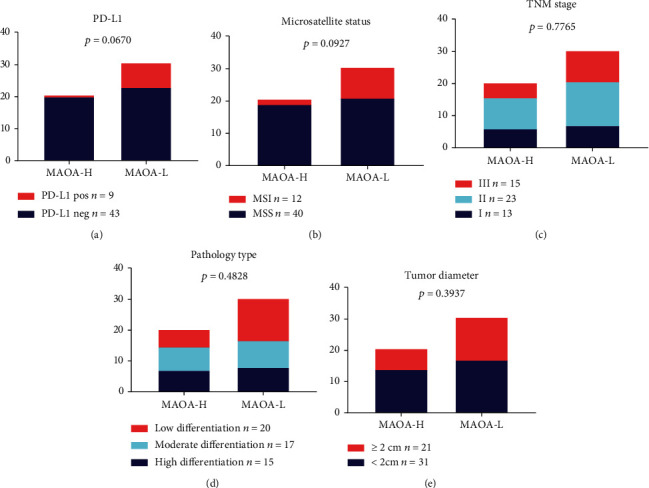
Correlation between MAOA expression and the patient clinicopathological characteristics. Patients with low MAOA expression had a larger ratio of PD-L1 positivity and MSI than those with high MAOA expression. Regarding the TNM stage pathology type and tumor diameter, no significant trends were found between the high- and low-expression groups.

Finally, the clinicopathological characteristics of gastric cancer patients, such as PD-L1 expression, the microsatellite status, TNM stage, pathology type, and tumor diameter, were analyzed in 52 patients with MAOA expression. Twenty-one patients showed high MAOA expression, and 31 showed low MAOA expression. The low MAOA-expressing patient cohort showed a larger ratio of PD-L1-positive patients than the high MAOA-expressing group. Eight patients in the MAOA low-expression group and 1 patient in the high-expression group had a PD-L1-positive status. The microsatellite status showed the same trend as PD-L1 in the low MAOA-expressing patient cohort because of the larger MSI ratio. However, the above difference was not significant (*p* = 0.0670 and *p* = 0.0927) (Figures 6(a) and 6(b)). Regarding the TNM stage, pathology type, and tumor diameter analysis, no significant differences were found between the high- and low-expression groups (*p* = 0.7765, *p* = 0.4828, and *p* = 0.3937) (Figures 6(c)–6(e)).

## 5. Discussion

Monoamine neurotransmitters include catecholamines and indoleamines. Catecholamines include dopamine (DA), norepinephrine (NE), and epinephrine (E); indoleamines mainly comprise serotonin (5-HT). In the present study, the expression levels of DA, NE, E, and 5-HT in gastric normal and tumor tissues were detected. NE was highly expressed in gastric cancer tissue, while the other monoamine neurotransmitters were not significantly different. Subsequently, NE synthetase enzymes tyrosine hydroxylase (TH), dopa decarboxylase (DDC), and dopamine-*β*-hydroxylase (DBH), degrading enzymes monoamine oxidase A and B (MAOA and MAOB), and catechol-O-methyl transferase (COMT) were analyzed in TCGA database of gastric cancer. Both MAOA and MAOB showed low expression in gastric cancer tissue compared with that in normal tissue. NE is also known as a stress neurohormone derived from the amino acid tyrosine and released primarily from the adrenal medulla and sympathetic nerves. According to previous studies, growing evidence indicates that the neurotransmitter NE affects some cancer types. Chronic use of beta-blocking drugs results in lower recurrence, progression, or mortality of breast cancer [12, 13] and malignant melanoma [14, 15]. Preclinical studies have shown that manipulating the levels or receptors of NE with drugs affects experimentally induced cancers. The mechanism is considered to be the following: NE may affect cancer by interacting with molecular pathways already implicated in abnormal cellular replication, such as the P38/MAPK pathway, or via oxidative stress [16].

NE is profoundly implicated in multiple biological behaviors of cancers, including cancer cell survival, proliferation, resistance to apoptosis, invasion, metastasis, and angiogenesis, in the tumor microenvironment [17–19]. To explore its potential pathway in gastric cancer, the NE-degrading enzymes MAOA and MAOB were divided into the high- and low-expression groups to find potential related signaling pathways in our study. The glycolysis pathway and interferon-*γ* pathway were relatively active in both the MAOA and MAOB low-expression groups. Neurotransmitters regulating glucose metabolism have been reported in recent years. For example, 5-HT promotes the growth of pancreatic cancer cells under metabolic stress by regulating aerobic glycolysis [11]. Another study found that aerobic glycolysis occurs during brain activation and is regulated and influenced by NE in astrocytic metabolism [20], indicating that neurotransmitters play an important role in aerobic glycolysis in normal cells or tumor cells. Another study on the effect of NE on the angiometabolic switch in prostate cancer showed that NE binding to its receptors activates an “angiometabolic switch,” which changes how cells metabolize glucose. The stimulation of NE released from nerves promotes endothelial cells to maintain the use of glycolysis, enabling prostate cancer to rapidly progress from a low-grade precancerous stage to a high-grade malignant stage [21]. In our study, whether at the cell line level of gastric cancer or PET-CT imaging findings for cancer patients, the neurotransmitter NE was closely related to glycolysis. Therefore, we speculate that the neurotransmitter NE may regulate glycolysis in gastric cancer cells, and further experiments are needed to confirm this hypothesis.

Next, the relationship between the neurotransmitter NE expression level and immune characteristics was explored in this study. In previous studies on melanoma, NE upregulates the expression of the protumorigenic cytokines VEGF, IL-8, and IL-6 and metalloproteases in tumor cell lines [22–24]. In ovarian cancer, NE increases the production of IL-8, which stimulates the growth of tumor cells [25]. Therefore, NE has been shown to influence inflammatory immune cells in cancers. NE receptors beta-adrenergic receptors (*β*-ARs) are present in many types of immune cells, such as helper and T suppressor lymphocytes, B lymphocytes, NK cells, macrophages, and dendritic cells [26]. Additionally, the *β*-AR-mediated hormone signaling pathway regulates macrophage differentiation [27, 28] and antigen-specific T cells [29]. Blocking the *β*-AR signaling pathway or chemical sympathectomy effectively abolishes the effects of the NK cell-enriched environment and attenuates the antitumor effect [30]. Given that NE is closely related to the regulation of numerous immune cells in the tumor immune microenvironment, we conducted an analysis of relevant indicators for the clinical evaluation of immunotherapy results with its degrading enzyme MAOA. However, in this study, for patients with low MAOA expression, the positive rate of PD-L1 and proportion of MSI were higher, and the difference between the two groups was not statistically significant. A larger sample size may be required to verify the correlation. Collectively, these findings suggest the emerging roles of NE and its *β*-AR signaling pathway in immune therapy evaluation. Additionally, if patients with low MAOA expression have a poor survival prognosis and a good response to immunotherapy, the prognosis of these patients will be greatly improved.

A close relationship exists between tumor metabolism and immunotherapy, and the effect and response of immunotherapy can be enhanced by interfering with the metabolism of tumor cells or immune cells [31, 32]. In conclusion, we detected the expression of monoamine neurotransmitters in gastric cancer tissues and adjacent normal tissues and found that NE is highly expressed in gastric cancer tissues. Next, we analyzed the expression of NE synthetase and degrading enzymes in TCGA database and learned that the two degrading enzymes MAOA and MAOB have apparent expression differences. Through GSEA, we found that the enrichment of the MAOA and MAOB high- and low-expression groups both contained glycolysis and interferon gamma pathways. After that, we analyzed the correlation between NE expression and glycolysis activity and found that high NE expression in gastric cancer cells or tissues may promote glycolysis. The exact mechanism requires further verification. High NE expression in gastric cancer tissue has a certain relationship with PD-L1 positivity and MSI, although no significant difference was found because of the limited sample size. In the future, the sample size can be increased to clarify the correlation between NE expression and the effect of immunotherapy to provide more reference indicators to evaluate immunotherapy effects. The mechanism also requires further experimental investigation and verification.

## Data Availability

TCGA database analyzed during the current study is available on The Cancer Genome Atlas website (https://cancergenome.nih.gov/) and GSEA (http://www.gsea-msigdb.org/gsea/index.jsp). Other data used to support the findings of this study can be requested from the corresponding author by email (microzhaoenhao@163.com).

## Supplementary Materials

Supplementary materials Supplementary Table 1: primers used in this study.
