# Supplementary material for: Norepinephrine Enhances Aerobic Glycolysis and May Act as a Predictive Factor for Immunotherapy in Gastric Cancer
Source: J Immunol Res. 2021 Mar 27;2021:5580672. doi: 10.1155/2021/5580672 (PMC8019630; doi:10.1155/2021/5580672)
Supplement: Supplementary materials — Supplementary Table 1: primers used in this study. [file 5580672.f1.docx]

**Supplementary Table1 Primers Used in This Study**

| Gene symbol | Forward sequence (5’-3’) | Reverse sequence (5’-3’) |
| --- | --- | --- |
| GLUT1 | ATTGGCTCCGGTATCGTCAAC | GCTCAGATAGGACATCCAGGGTA |
| HK2 | AGCCCTTTCTCCATCTCCTT | GCTTGCCTACTTCTTCACGG |
| PDK1 | CTGTGATACGGATCAGAAACCG | TCCACCAAACAATAAAGAGTGCT |
| PKM | ATGTCGAAGCCCCATAGTGAA | TGGGTGGTGAATCAATGTCCA |
| GPI1 | CAAGGACCGCTTCAACCACTT | CCAGGATGGGTGTGTTTGACC |
| PFKL | GGTGCCAAAGTCTTCCTCAT | GATGATGTTGGAGACGCTCA |
| LDHA | ATGGCAACTCTAAAGGATCAGC | CCAACCCCAACAACTGTAATCT |
| GAPDH | CTGGGCTACACTGAGCACC | AAGTGGTCGTTGAGGGCAATG |
| ALDOA | AACTTTCCTCTGCCTAGCCC | GTACAGGCACAGTCGCAGAG |
| ENO1 | GCCGTGAACGAGAAGTCCTG | ACGCCTGAAGAGACTCGGT |
| PGAM2 | AGAAGCACCCCTACTACAACTC | TCTGGGGAACAATCTCCTCGT |
| PGK2 | AAACTGGATGTTAGAGGGAAGCG | GGCCGACCTAGATGACTCATAAG |
| TPI1 | AGCTCATCGGCACTCTGAAC | CCACAGCAATCTTGGGATCT |
| MAOA | GAATCAAGAGAAGGCGAGTATCG | GGCAGCAGATAGTCCTGAAATG |
| MAOB | GGCGGCATCTCAGGTATGG | GGTCTCCAATCCTAGCTCCTTG |
